# Diet patterns associated with cognitive decline: methods to harmonize data from European and US cohort studies

**DOI:** 10.3389/fnut.2024.1379531

**Published:** 2024-03-21

**Authors:** Amaia Ayala-Garcia, Natalia Soldevila-Domenech, So-Yun Yi, Rafael de la Torre, Lyn M. Steffen, for the IAFNS Retrospective Harmonization Expert Working Group, Courtney K. Blackwell, Christina Khoo, Maxwell Armand Mansolf, Emilio Ros, Linda Snetselaar, Tina Wey

**Affiliations:** ^1^Integrative Pharmacology and Systems Neurosciences Research Group, Neurosciences Research Program, Hospital del Mar Research Institute (HMRI), Barcelona, Spain; ^2^Division of Epidemiology and Community Health, University of Minnesota School of Public Health, Minneapolis, MN, United States; ^3^Department of Medicine and Life Sciences, Universitat Pompeu Fabra, Barcelona, Spain; ^4^CIBER de Fisiopatología de la Obesidad y Nutrición, Instituto de Salud Carlos III, Madrid, Spain

**Keywords:** diet, cognition, harmonization, meta-analysis, protocol, longitudinal data

## Abstract

The impact of dietary intake on cognitive outcomes and dementia prevention is a topic of increasing interest. Meta-analyses of observational studies, mostly conducted within US and European populations, have reported benefits of healthy diet patterns on cognitive performance, but results from individual studies have been inconsistent. These inconsistencies are likely due to the diverse methodology used in studies, including different diet and cognitive function assessment instruments, follow-up periods, and analytical methods, which make drawing conclusions relevant to dietary guidance challenging. The objective of this project is to describe a protocol to conduct a retrospective harmonization study on dietary intake and cognitive health using data from European and US studies. The recommendations resulting from the project can be used to support evidence-based synthesis for future iterations of the Dietary Guidelines for Americans or other population-based dietary guidance. Additionally, this study will serve as a harmonization guide for future research on the relationship between diet patterns and cognition. The approach outlined ultimately aims to optimize resources and expedite research efforts for dementia prevention.

## 1 Introduction

Over the past few decades, there has been increasing interest in exploring the impact of dietary intake on cognitive outcomes and dementia prevention (1). Numerous high-quality systematic reviews and meta-analyses of observational studies, mostly conducted within US and European populations, have evaluated associations between several diet patterns and cognitive functioning or dementia prevention. Most of these diets emphasize the consumption of vegetables, fruits, and whole grains (2, 3). However, the results were inconsistent for individual studies of cognitive function and diet patterns, including the Mediterranean diet (4, 5), the Dietary Approach to Stop Hypertension (DASH) diet (6, 7), the Mediterranean-DASH diet Intervention for Neurodegenerative Delay (MIND) diet (8, 9), or the anti-inflammatory diet pattern (10).

Despite promising preliminary results of meta-analyzing healthy diet patterns relative to cognitive outcomes, drawing conclusions relevant to dietary guidance remains challenging. The complexity arises from the inherent difficulties in combining and evaluating existing evidence given the heterogeneity in the study populations and the cognitive and dietary assessment methods employed, the different diet patterns and cognitive outcomes examined, and the differential length of the follow-up or timing of outcome assessment across studies (11). Thus far, there has been a lack of standardizing dietary intake and cognitive test data prior to data analysis (12–14). This hinders the interpretation of the study results and, consequently, limits the effectiveness of systematic reviews and meta-analyses in guiding dietary recommendations (15).

These challenges were highlighted by members of the 2020–2025 Dietary Guidelines Advisory Committee (DGAC) comprised of nutrition and public health experts (11). The DGAC assessed the current body of nutrition science, offering independent, science-based counsel to the US Departments of Health and Human Services (HHS) and Agriculture (USDA) during the formulation of the 2020–2025 Dietary Guidelines for Americans (11). However, the DGAC characterized the available evidence about diet patterns and risk of age-related cognitive impairment and/or dementia as limited and were unable to generate a recommendation (11). Recognizing that the issue of methodological disharmony is likely to persist in the 2025–2030 DGAC’s and future such reviews, one potential resolution involves developing a systematic approach to address this longstanding challenge within the literature.

One such approach is to pool individual participant data (IPD) collected in multiple large-scale observational cohort studies and clinical trials (16). IPD meta-analysis is considered the gold standard approach to evidence synthesis (17). Unlike most systematic reviews, it does not rely on aggregate data extracted from journal publications. Rather, the original data on each individual participant are sought from each eligible study. With larger sample sizes resulting from the pooled analysis it might be possible to enhance the statistical power to detect associations not evident in small cohorts. Moreover, analyses are standardized across studies, including variable definitions, statistical methods, and covariates, which reduces variation and potential bias in estimates. However, data cannot be pooled without careful harmonization. IPD harmonization allows integrating information from different studies while ensuring compatibility and inferential equivalence (comparability) across them.

Under the guidance of an Expert Group participating in this project and aligned with previously described guidelines (18–21), we propose to document the procedures for conducting a retrospective harmonization study about dietary intake and cognitive health using data from several European and US studies. Here, we describe our study protocol, including criteria for the selection of studies to answer our study question, define study exposures and outcomes, and describe the process we will take to harmonize these data. In addition, we briefly describe the IPD meta-analytical approach to assess the association of diet pattern intake with cognitive performance using the harmonized datasets.

## 2 Methods

For the purpose of this paper, we use the following study question to provide direction for harmonizing and meta-analyzing data from appropriate studies: *What are the associations of diet pattern intake with cognitive decline or incidence of mild cognitive impairment (MCI)?*

**Figure 1:**
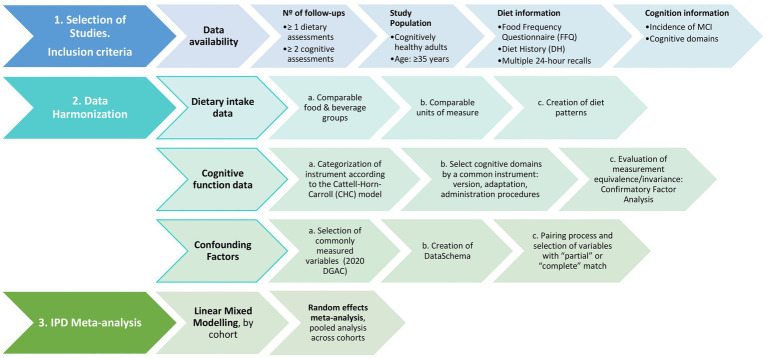
Schematic study protocol to assess the association between diet patterns and cognitive decline.

IPD harmonization necessitates a rigorous evaluation and documentation of studies participating in the exercise and a meticulous process to harmonize and integrate study-specific data under a common format. The research questions and objectives guide the harmonization procedures (18), which will be conducted as specified in Figure 1.

### 2.1 Selection of studies

Selection of studies to address the study question will be those that meet the following criteria:

1. Data should be accessible, such as datasets available from the BioLINCC data repository that is managed by the National Institutes of Health/National Heart, Lung and Blood Institute in the US and datasets from well-established European repositories (e.g., Dementia Prevention UK data portal). Data should also be representative of different diets and populations across Europe and US.
2. The study design may be a prospective cohort study and/or clinical trial with at least 2 follow-up examinations that include at least one assessment of usual dietary intake at baseline, and more than one measure of cognitive performance collected through validated questionnaires. The reason behind these criteria is that at least two measures of cognitive performance at different points in time are needed to be able to assess the potential impact of a diet pattern on cognitive changes and incidence of MCI. If participants underwent multiple diet assessments at different time points, the dietary data from those assessments will be averaged to generate a single dietary profile for each participant. This averaging process aims to mitigate the effects of random variability and measurement error, providing a more stable representation of participants’ dietary intake. The choice of which assessments to average will be based on various factors, including the timing of assessments, consistency of responses, and validity of measures. Sensitivity analyses will be conducted to assess the robustness of results to different combinations of assessments.
3. The study population includes cognitively healthy adults living in the community (not assisted living or a nursing home) and at least 35 years old at baseline. Study participants with a diagnosis of MCI or dementia at baseline will be excluded. The interaction effect of several comorbidities or lifestyle factors (e.g., cardiovascular function, obesity, depression, diabetes, or physical activity) in the association between diet patterns and cognitive change will be assessed, including sensitivity analyses with these groups. Several models will be developed, including minimally to fully adjusted models, for example, model 1: adjusting for demographic characteristics and energy intake; model 2 adjusting for model 1 plus lifestyle factors; and model 3 adjusting for model 2 plus comorbidities, if the interaction tests were not significant. All the covariates will be harmonized.
4. The study has collected data to harmonize and derive the following variables:

- The exposure variable will be diet patterns, such as alignment with the Healthy Eating Index (HEI) (22), the EAT-Lancet pattern (23), Mediterranean diet pattern (24)–adapted score for European and US studies (25), and/or the ‘*a posteriori*’ diet pattern derived by principal components analysis (26). The scores will be derived from daily intakes of food group and nutrient data assessed via food frequency questionnaire (FFQ), Diet History (DH), or multiple 24-h recalls that were interviewer-administered (self-reported dietary intake constitutes an exclusion criteria). The diet assessment should be conducted prior to or simultaneously with the first cognition assessment.
- The outcome variables include assessments of cognitive performance at a minimum of two time points to measure cognitive change and incidence of MCI as per cut-offs of cognitive screening tests available in the studies (i.e., Mini-mental state examination (MMSE) or Montreal Cognitive Assessment (MoCA). Assessment of cognitive function may include a list of instruments measuring (i) verbal memory, (ii) verbal fluency, and (iii) executive function as specific cognitive domains, which could vary between studies. All cognitive outcomes (MCI, verbal memory, verbal fluency, and executive function) will be assessed separately.
- Confounding factors known to be related to cognition and/or dietary intake will be included (11, 19, 27). In statistical models, variables considered by the DGAC will be included: age, ethnicity, education, socioeconomic status, smoking, physical activity, anthropometry, and alcohol intake (27). Moreover, an *a priori* larger list of confounders reported in the literature will be considered, such as blood pressure, lipids, glucose, medication use, and others (28). The final confounder list will include, at the very minimum, the DGAC confounder list.
- All analyses will be stratified by sex.

### 2.2 Data analysis

#### 2.2.1 Harmonizing dietary intake data

Our final exposure variables will be diet patterns. To create them, the dietary data will be harmonized into (i) comparable food and beverage groups, such as fruit, vegetables, dairy, meat, sugar sweetened beverages, candy, etc., (ii) comparable units of measure, including servings/day and/or grams/day (29, 30). As a third and last step, diet patterns will be created with the harmonized data. These steps are explained in more detail below.

In order to harmonize dietary information into comparable food and beverage groups, information from DH, multiple 24-h recalls and FFQ will be used. Food subgroups obtained from a DH and multiple 24-h recalls are typically similar and more flexible to combine into a larger food subgroup than food categories listed on a FFQ. Therefore, the DH and 24-h recall food groups will be tailored to those of the FFQ. Some food categories listed on a FFQ include mixed dishes, such as pizza, fast food sandwiches, chicken, vegetable, and rice mixed dish (or casserole). These foods must be disassembled into the component parts. For example, a cheese pizza consists of refined grain crust, tomato sauce, and cheese. The component parts will be assigned to the respective food subgroup. Another example is where cheese, meat, and vegetable pizza are included in one food category; therefore, components for cheese, meat, and vegetables will be included in one recipe and components assigned to the respective food subgroup. Finally, major food groups will also be created by summing similar food subgroups.

The nutrients reported in diet studies are usually similar, however, units may differ. For example, energy intake may be reported as kilocalories or kilojoules; thus, it is necessary to convert one unit to the other. In addition, the output data from a diet assessment include servings or grams from each food group or food category consumed as well as nutrient intake. In the US, serving sizes for foods and beverages are standard, being based on USDA information (31). In Europe, portion sizes are reported in grams/day for each major food group.

Finally, diet patterns will be created using the harmonized dietary data. The final exposure variables for our study question are healthy diet patterns; for example, the HEI-2020, a score that represents diet quality (22). Food groups in servings/day as well as added sugars (grams/day) are components required for this score. Mediterranean diet pattern will also be assessed, and validated scores will be selected depending on the origin of the included studies (25). Another potential diet pattern of interest is the EAT-Lancet pattern, which was developed by experts from various fields, including health, agriculture, environmental sustainability, and political science, to examine the links between diet, human health, and the environment (32). This pattern requires grams/day of food group consumption, not servings/day of food intake, as well as daily intakes of added fats and added sugars (32). Also, some patterns include nutrients in the algorithm, e.g., added sugar, saturated fat, polyunsaturated fat, and monounsaturated fat, in addition to the food and beverage groups.

#### 2.2.2 Harmonizing cognitive function data

Cognitive measures across studies will be restricted to instruments that indicate a potential diagnosis of MCI, or that measure verbal memory, verbal fluency, and executive function. Decline in these cognitive domains often serves as the initial clinical indication of cognitive impairment and dementia due to Alzheimer’s disease (33, 34), and are commonly measured in neuropsychological evaluations.

To harmonize cognitive data a pre-statistical harmonization aimed at ensuring accurate and consistent inferences about cognitive health across studies (e.g., qualitative process involving a review of cohort characteristics and cognitive instruments) (21) will be carried out by categorizing cognitive tests measured in each study into cognitive abilities of interest, and assessing heterogeneity among common tests to determine comparability of tests across cohorts.

In order to determine cognitive abilities measured by cognitive instruments in each study, we will categorize each instrument according to the Cattell-Horn-Carroll (CHC) model (35), which is the most psychometrically established model of cognitive ability. Instruments measuring MCI, verbal memory, verbal fluency, and executive function according to the CHC model will be selected. As a rule of thumb, when investigating the measurement equivalence of overlapping measures within and across cohorts, we will prioritize cognitive tests that are identical. If identical tests are not available, we will focus on conceptually similar tests, and process these data to place them on comparable metrics (36).

Regarding the evaluation of potential sources of heterogeneity among common instruments, we will look at instrument version, instrument adaptation, administration procedures and component items. Data augmentation strategies will be applied if necessary to adjust for differences uncovered during the pre-statistical harmonization process, including alignment of coding procedures, winsorization, and equipercentile equating (21). To that end, we will conduct detailed exploratory analyses of test scores (e.g., dot plots and histograms, stem and leaf plots, tables of minima, maxima, medians, and means) by cohort for every test item presumed to be comparable across cohorts. Harmonization of cognitive measures will adhere to the guidelines provided by the Agency for Healthcare Research and Quality (19). First, we will apply simple monotonic linear transformations to place raw scores on comparable metrics (36). This approach is different from standardization (e.g., use of Z scores), as it only changes the absolute metric of a test, thus it allows the comparison of mean levels of a given variable across cohorts or assessment waves, and relies on comparability of the domain(s) measured by the harmonized tests (37). Then, a latent variable modeling approach will be used to estimate the quality of harmonized variables. We will test measurement equivalence of tests across assessment waves and cohorts. Each test will be conceptualized as an observable indicator of an unobserved (e.g., latent) general cognitive variable (e.g., verbal memory, verbal fluency and executive function) (19, 36).

Measurement equivalence or invariance will be tested by confirmatory factor analysis (CFA), by comparing a series of increasingly strict parsimonious models. More parsimonious or strict models allow a lesser number of parameters to vary over time for the same latent construct. Such parameters are factor loadings (representativeness of each item, labeled as λ), intercepts (mean levels of each item, labeled as τ) and residual variances (unexplained influences predicting item responses, labeled as ɛ). Levels of invariance range from configural that allows λ, τ and ɛ to vary across time, followed by metric invariance that constraints λ, scalar invariance that also constraints τ, and residual invariance that additionally constraints ɛ. If, after fitting equality constraints across cohorts/waves, we do not observe a worsening of absolute model fit (38), then the said level of measurement is judged to hold, and the parameters in question can be considered equivalent. Those tests/cohorts that may harm the assumption of measurement equivalence will be excluded from the analysis.

#### 2.2.3 Harmonizing confounding factors

Definition of potential confounding factors will be made based on a selection of commonly measured confounding factors which are known to influence cognitive function and/or diet (19), at the very minimum the ones selected by the DGAC in their recent protocol for the systematic review of the evidence on dietary patterns and risk of cognitive decline will be included: age, ethnicity, education, socioeconomic status, smoking, physical activity, anthropometry, and alcohol intake (11, 27).

Harmonization of potential confounders will include (i) pre-statistical harmonization using the Maelstrom Research approach (18, 39). For this purpose, we will first define a set of variables targeted for harmonization in a DataSchema and resolved on an *a priori* description of each variable in order to decide if information collected on each study can be combined in a pooled analysis. And (ii) pairing process to determine compatibility of each study’s data and each variable in the DataSchema on a three-level scale of matching quality: “complete,” “partial,” or ‘impossible” match which will be reported as part of the harmonization process. However, all variables of interest that are a “complete” or “partial” match to the DataSchema will be included in the final data analysis (19).

#### 2.2.4 IPD meta-analysis plan

Associations between diet patterns and cognitive performance will be analyzed using random effects IPD meta-analysis (40). Mixed models are widely used to analyze longitudinal data and are recommended to address missing data as well as to reduce non-random attrition bias. First, linear mixed models will be used to examine the associations of diet patterns and baseline cognitive performance, as well as cognitive decline (per year) on each outcome, separately for each cohort (40). Second, random effects IPD meta-analysis will be used to pool the cohort-wise linear mixed model results to obtain pooled estimates of effect sizes for each of the model terms. All analyses will be stratified by sex.

## 3 Discussion

Around the world, continuing gains in life expectancy coupled with declining fertility rates are producing deep changes in demographic profiles. Today, the world’s population is more than three times larger than it was in 1950 and, by the year 2050, it is estimated to increase by nearly 2 billion people (41). With advancing age, cognitive decline becomes evident, significantly affecting independent living and serving as a hallmark of Alzheimer’s disease and related, largely untreatable dementias. Identifying interventions with the potential to prevent or delay cognitive decline is therefore a critical global public health priority.

As diet has been identified as a modifiable risk factor for the prevention of dementia (42), institutions responsible for developing guidelines and dietary recommendations for the general population point to the need for high quality evidence to answer the long-standing question: What is the relationship between diet patterns and risk of cognitive decline? (27). As highlighted above, numerous attempts have been made to shed light on this question, but different study designs and approaches to measuring both diet patterns and cognitive function have hampered the ability to draw robust conclusions for making specific dietary recommendations. As a result, cognitive endpoints are increasingly being used as primary and secondary outcomes in nutrition research. Unfortunately, there is a lack of consistency in the cognitive tasks used across studies, as well as a lack of standardization in the way cognitive test data are analyzed and reported, which adds to the difficulty of interpreting cognition itself (13).

This study protocol aims to establish the necessary criteria and analysis plan to address the lack of high-quality evidence by harmonizing the existing evidence by a multidisciplinary team of experts in the field of nutrition and cognition. To conduct an IPD meta-analysis from numerous prospective studies, all variables, including the exposure, outcome, and confounding factors, should be comparable across studies. Thus, the main objective for our study protocol was to describe the harmonization process, including the description of the instruments used to assess dietary intake and cognitive function as well as the confounding factors, but also the output. Another important consideration for obtaining robust findings is timing of assessment (43). While in prospective studies of diet and cognition there are at least one baseline and one follow-up assessment of cognitive outcomes, diet assessment (exposure) is typically done at baseline, even when follow-up spans 20 years. This is a critical limitation of many cohort studies. In nutritional epidemiology, repeated measurements of exposure are highly informative because they best represent long-term diet and minimize within-subject variation (44). For this reason, selection of studies with several diet measurements during follow-up will be prioritized.

IPD meta-analysis increases the validity of results and also aligns with the efficient use of resources, as accessible or public data is generally ready to be used and understood by the research community. However, it also poses with limitations as data has not been collected for the purpose of the study.

This protocol will be followed by a results paper with detailed information on all the pitfalls encountered, from the initial stages and data request to the harmonization process and interpretation of the results. Most importantly, the results will help to develop dietary recommendations for the prevention of cognitive decline in middle-aged and older adults that might be considered by future Dietary Guidelines for Americans Advisory Committees in their deliberations. It will also serve as a harmonization guideline for future studies investigating the relationship between diet patterns and cognition, and it is expected to enhance information dissemination on existing studies, crucial for optimizing resources and accelerating dementia prevention research.

## Author contributions

AA-G: Methodology, Writing – original draft, Writing – review & editing. NS-D: Methodology, Writing – original draft, Writing – review & editing. S-YY: Writing – original draft, Writing – review & editing. RT: Conceptualization, Funding acquisition, Project administration, Writing – original draft, Writing – review & editing. LS: Conceptualization, Funding acquisition, Project administration, Writing – original draft, Writing – review & editing.

## Member of the IAFNS Retrospective Harmonization Expert Working Group

Courtney K. Blackwell, PhD, Northwestern University, ckblackwell@northwestern.edu; Christina Khoo, PhD, Ocean Spray Cranberries, ckhoo@oceanspray.com; Maxwell Armand Mansolf, PhD, Northwestern University, maxwell.mansolf@northwestern.edu; Emilio Ros, MD, PhD, IDIBAPS, Barcelona, Spain, emilirosrahola@gmail.com; Linda Snetselaar, PhD, RD, University of Iowa, linda-snetselaar@uiowa.edu; Tina Wey, PhD, Maelstrom Research, McGill University Health Centre, twey@maelstrom-research.org.
